# Lambda-vector modeling temporal and channel interactions for text-independent speaker verification

**DOI:** 10.1038/s41598-022-22977-5

**Published:** 2022-10-28

**Authors:** Guangcun Wei, Hang Min, Yunfei Xu, Yanna Zhang

**Affiliations:** 1 College of Intelligent Equipment, Shandong University of Science and Technology, Taian, 271019 Shandong China; 2 College of Computer Science and Engineering, Shandong University of Science and Technology, Qingdao, 266590 Shandong China

**Keywords:** Engineering, Mathematics and computing

## Abstract

Most of the current excellent models in speaker verification are ResNet-based deep models and attention-based models. These models have a general weakness, which is the large number of parameters and high hardware requirements. On the other hand, many deep structures only generate embedding features from the features extracted by the last frame-level layer, which causes shallow features and channel-related features to be ignored. To solve these problems, this paper proposed a shallow speaker verification model based on Lambda-vector, its main structure is composed of three Lambda-SE modules. The module extracts long-distance dependencies between frame-level features and channel-related interaction information to enhance representation of features. Meanwhile, so that adequately mine the information in deep and shallow features, the model introduces multi-layer feature aggregation to fuse the features of different frame-level layers together. It can increase the detailed information in the deep features and improve the model's ability to represent complex information. The experimental results on the public datasets Voxceleb1 and Voxceleb2 show that the model has more stable training speed, fewer model parameters, and better identification performances than baseline models.

## Introduction

Deep learning has been widely used in identity and biomedical identification^1^. Speaker verification is a biometric technology, which is the task of verifying the authentic identity of a person based on his or her voice features^2^. It has become one of the key technologies in banking, finance, e-commerce, judicial forensics, and other fields. According to the different identification objects, it can be divided into text-dependent and text-independent types. Text-dependent speaker verification requires that the speech content of the registered utterance and the test utterance are identical, while text-independent does not require the text content of utterances.

The algorithms of speaker verification are broadly classified into traditional algorithms and deep learning-based algorithms. Traditional algorithms include hidden Markov model (HMM)^3^, gaussian mixture model (GMM)^4^, joint factor analysis (JFA)^5^, identify-vector (i-vector)^6^, etc. These models are generally based on mathematical derivation of shallow structures, which process the voice signal at a lesser level and cannot completely capture structural information and deep features of utterances.

**Figure 1:**
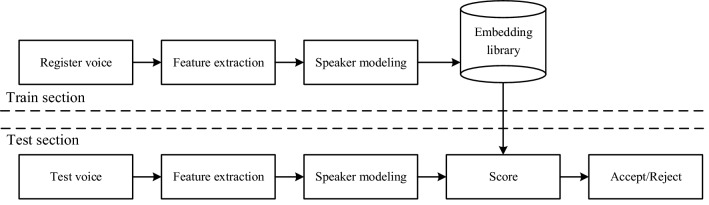
The framework of speaker verification system based on speaker embedding.

In recent years, deep learning has been rapidly developing in the field of speaker verification, and most of the advances in text-independent speaker verification systems can be attributed to deep neural networks (DNN) models. DNN-based systems can be divided into two categories: end-to-end and speaker embedding-based. End-to-end systems^7–11^ directly verifies the input a pair of utterances to produce similarity score. The method consists of a single neural network structure that uses a joint optimization approach to train the various components of the system, including the process of confirming the evaluation. The speaker embedding-based systems are shown in Fig. 1, which are stage-wise and normally consist of three parts:

- Feature extraction: This step converts the original utterances into feature vectors and takes out the redundant information in the speaker's utterances.
- Speaker modeling: The main role is to extract information from frame-level features, aggregate them to segment-level features, and map to speaker embeddings.
- Score: It is calculated that the similarity between the registered and the test utterances, primarily includes two methods: cosine distance score and probabilistic linear discriminant analysis^12^.

The strategic process to the embedding-based systems is speaker modeling, which is the extraction of embedding features. A novel DNN-based speaker modeling method, d-vector^13^, was first proposed in 2014. The method sets the real speaker identity as the label of the training frame to train a DNN model for frame-level speaker verification. Thereby it transformed the model training into a classification problem. X-vector^14,15^ is an important evolution of d-vector, which utilizes a statistical pooling mechanism to convert variable-length input into fixed-dimensional speaker embedding features. With the introduction of deep residual networks, it has also been widely used in the field of speaker verification to extract frame-level features.

Early speaker embeddings were extracted from classification networks that averaged frame-level features, they were considered equally important and failed to target features that represent speaker identity. The attention mechanism^16,17^ solved this problem, which makes the speaker embeddings focus on the representative frame-level features^7^. It provides importance-weighted standard deviations and weighted averages to emphasize unique features^18^, resulting in a more discriminative representation of long-range features. The proposal of Transformer^19^ has immensely facilitated the application of self-attention in speaker verification. Self-attention is a variant of the attention mechanism, which solves the interaction problem of frame-level features by calculating the mutual influence between adjacent frames. It reduces the dependence on external information and is expert in capturing the internal correlation of data or features. Pooyan et al. propose an end-to-end speaker embedding extractor^20^ based on self-attention and transformer encoders. Multi-view self-attention^21^ balances the ability to capture global dependencies and modeling by using sliding windows of different sizes for each attention head.

Self-attention has the property of global computation and can establish long-range dependencies. However, the unrestricted computation of self-attention produces an attention graph that consumes a large amount of memory resources. In high-resolution images, the computational complexity of self-attention is quadratic to the image size^22^, which limits its application in many vision tasks and long-sequence tasks such as speaker verification. And models^15,20,21^ built using self-attention or convolutional layers only captures time-related features, which overpass channel-related features. Under the condition of noise, different types of noise will affect the channel information of voice signal, but these models cannot capture them. Even more, these models extract only the deep features in the last frame-level layer and ignore the frame-level extractor to generate shallow features.

To reduce the number of model parameters and to extract shallow features and channel-related features, we propose a straticulate speaker verification network and name it Lambda-vector. It has constructed the Lambda-SE module by Lambdalayer^23^ and squeeze-and-excitation block (SE-Block)^24^. Lambdalayer is a new type of attention that captures long-range interactions without expensive attention maps. SE-Block explicitly modeling the interdependencies among the channels of its convolutional features, and then enhances or suppresses different channels for different tasks. We combine them together via convolution computation and residual connection to model long-distance dependencies between frame-level features, and extract channel-related interaction information. Multi-layer feature aggregation is designed to integrate frame-level features of different layers, which has increased the detailed information in the deep features and improved the model's ability to represent complex information.

## DNN speaker verification systems

Currently, the two most popular used DNN models for speaker verification are based on x-vector^14,15^ and ResNet^25^ architectures, which have derived many modifications^26–31^.

### X-vector

X-vector takes Mel frequency cepstral coefficients (MFCCs) as input to extract speaker embeddings and capture the interaction between contiguous frame-level features. The model is composed of three main parts: frame-level layer, segment-level layer, and statistical pooling layer that connects the frame-level and segment-level layers.

The frame-level layer is constructed by a five-layer time delay neural network (TDNN). TDNN is a special form of convolutional neural network (CNN) and belongs to a one-dimensional CNN. The layer non-linearly maps the input MFCCs to obtain frame-level features.

The statistical pooling layer that aggregates the output vectors from the last TDNN layer, calculate their mean and standard deviation, and perform splicing to convert the variable-length frame-level features into fixed-length segment-level features.

The third part is the segment-level layer, in which some fully connected hidden layers are stacked. The bottleneck layer is designed to have a smaller number of units, thereby mapping the information output by the pooling layer into low-dimensional embeddings. In the training phase, the features of the last fully connected layer are normalized by softmax function, and each softmax node corresponds to a speaker label. In the testing phase, the output features of the bottleneck layer are selected to score and verify the speaker identity in the back end.

### ResNet

Ordinary DNN will saturate and degrade rapidly with the increase of network depth. ResNet^25^ is proposed to alleviate the problem of increasing training difficulty caused by the deepening of network layers. It adds shortcut connections and identity mappings on the basis of feedforward neural networks to reduce the occurrence of overfitting. ResNet-based model structure is parallel to x-vector, which mainly includes three parts: ResNet, pooling layer, and segment-level layer. The function of ResNet likes the frame-level layer in x-vector, which is to extract frame-level features.

ResNet is composed of many stacked residual blocks (Res-Blocks), and the Res-Block in the widely used ResNet34 consists of two convolutional layers. Identity mapping is used to map the input vector of each Res-Block to the output vector. The expression defined by Res-Block in ResNet34 is shown in Eq. (1):

1 $$\begin{array}{*{20}c} {\rho = F\left( {x, W_{i} } \right) + x} \\ \end{array}$$

where $x$ and $\rho$ are the input feature vector and output feature vector respectively, ${W}_{i}$ is the learnable weight, $F(x,{W}_{i})$ is the output of the residual mapping, and $F+x$ is done by short connection and element-wise addition.

## Proposed method

**Figure 2:**
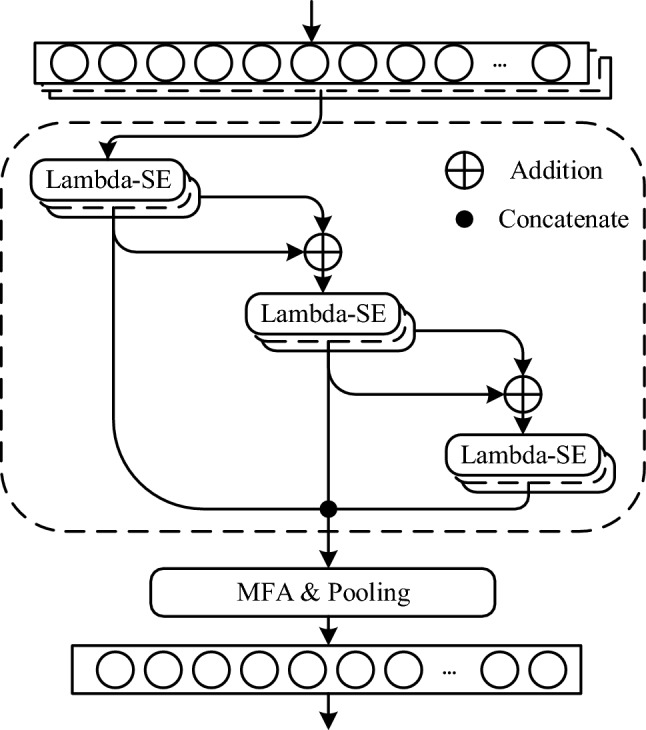
Graphic illustration of Lambda-vector. Multi-layer rectangles represent frame-level features. Single-layer rectangle represents segment-level features. The dashed rectangle represents the extraction process of frame-level features.

The overall framework of proposed model is shown in Fig. 2, which is composed of Lambda-SE modules, multi-layer feature aggregation, attentive statistics pooling and other structures. The detailed design is shown in Table 1. We first use a convolutional layer to increase the dimension of feature vectors. Conv1 converts 80-dimensional MFCCs into 256-dimensional feature vectors. After conv1 operation, a ReLU activation function and a batch normalization were added to the model. And then extract frame-level features through three Lambda-SE modules. The module is set as the main structure of the Lambda-vector model. To ensure multi-layer feature aggregation, the input and output of each Lambda-SE module are designed to be 256-dimensional. The concatenate operation concatenates the features in the temporal dimension to obtain 768-dimensional output. The role of conv2 is similar to that of conv1. The global layer concatenates features with their mean and standard deviation. Attentive statistics pooling converts 4608-dimensional frame-level features to 3072-dimensional segment-level features. Finally, the features are mapped to 512-dimensional speaker embeddings through a fully connected layer.

**Table 1:** Detailed design of proposed Lambda-vector model.

Level	Layer	Input size	Output size
Frame-level	Conv1	(80, N)	(256, N)
	ReLU + BN	(256, N)	(256, N)
	Lambda-SE1	(256, N)	(256, N)
	Lambda-SE2	(256, N)	(256, N)
	Lambda-SE3	(256, N)	(256, N)
MFA and pooling	Concatenate	3 × (256, N)	(768, N)
	Conv2	(768, N)	(1536, N)
	Global	(1536, N)	(4608, N)
	ASP	(4608, N)	(3072)
	BN	(3072)	(3072)
Segment-level	FC	(3072)	(512)
	BN	(512)	(512)

*N* number of input frame-level features, *BN* batch normalization, *Concatenate* concatenate in the temporal dimension, *ASP* attentive statistics pooling, *FC* fully connected layer.

### Lambda-SE module

**Figure 3:**
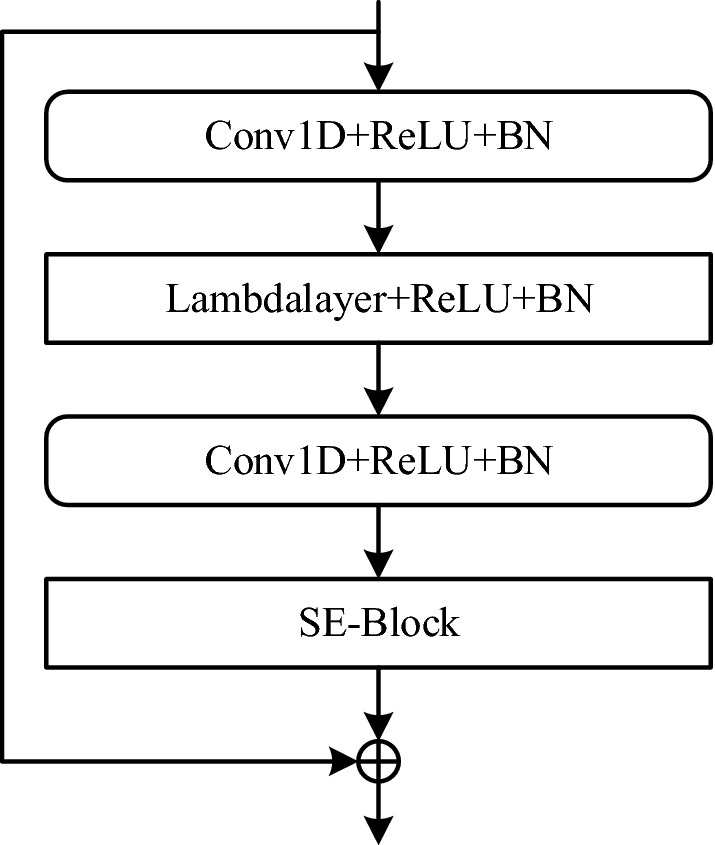
The Lambda-SE module of the Lambda-vector architecture.

The success of SE-Block shows that channel-dependent attention can enhance the feature expression ability. We believe that the combination of temporal attention-based Lambdalayer and SE-Block is beneficial to well excavate the representative characteristics of speakers. The structure of Lambda-SE is shown in Fig. 3, it consists of two one-dimensional convolutional layers, a Lambdalayer, and a SE-Block. In addition, each convolutional layer is subjected to ReLU function and batch normalization, which have non-linear feature conversion capabilities. Lambdalayer is used to extract the relationship between time-related frames, and SE-Block focuses on channel-related information.

In order to reduce the model parameters and make the network have more non-linear features, the convolutional layer before Lambdalayer to reduce the feature dimension, and then uses another convolutional layer to increase the data dimension. So that the extracted features and the input features dimensions keeping it consistent are convenient for multi-layer feature aggregation. This is followed by a SE-Block to model channel-related interactions. The final output of the Lambda-SE module is formed by adding the skip connection input and the output of the SE-Block.

#### Lambdalayer

Lambdalayer computes the output through a linear function ${R}^{\left|k\right|}\to {R}^{\left|v\right|}$, i.e., a matrix ${\lambda }_{n}\in {R}^{\left|k\right|\times \left|v\right|}$, which avoids huge attention maps. Lambdalayer computes keys $K$ and values $V$ by linearly projecting the context and producing normalized keys $\overline{K }$ by softmax operation. The matrix ${\lambda }_{n}$ is obtained by aggregating values $V$ using normalized key and position embeddings ${E}_{n}$.

2 $$\begin{array}{*{20}c} {\lambda_{n} = \overline{K}^{{\text{T}}} V + E_{n}^{{\text{T}}} V \in R^{\left| k \right| \times \left| v \right|} } \\ \end{array}$$

The query ${q}_{n}\in {R}^{\left|k\right|}$ is obtained from the input ${x}_{n}$ through a learnable linear projection. The columns of the lambda matrix are contextual features, which are aggregated by content and position. Making use of the lambda and then dynamically assign these features based on the query to generate the Lambdalayer's output $y_{n}$.

3 $$\begin{array}{*{20}c} {y_{n} = \lambda_{n}^{{\text{T}}} q_{n} \in R^{\left| v \right|} } \\ \end{array}$$

#### SE-block

**Figure 4:**
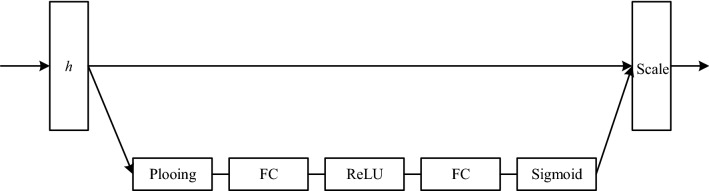
The SE-Block of the Lambda-SE architecture. $h$ represents the input frame-level features. Scale represents the channel-related weights.

The basic structure of the SE-Block is shown in Fig. 4. It first aggregates the features of the spatial dimension through the squeeze operation to obtain a channel descriptor, which includes the global distribution of channel features, and transmits the global distribution information to the excitation block. The squeeze operation is defined as:

4 $$\begin{array}{*{20}c} {z = \frac{1}{T}\mathop \sum \limits_{t}^{T} h_{t} } \\ \end{array}$$

where ${h}_{t}$ is the input frame-level feature, $t$ is the time step, and $z$ is the average vector of frame-level features over the entire time dimension. Then, through the excitation operation, the features of each channel are learned through the channel-dependent self-gating mechanism, and the descriptor in $z$ is used to calculate the weight of each channel. The excitation operation is defined as:

5 $$\begin{array}{*{20}c} {s = \sigma \left( {W_{2} f\left( {w_{1} z} \right)} \right)} \\ \end{array}$$

where $f(\cdot )$ is the ReLU non-linear activation function and $\sigma (\cdot )$ is the sigmoid function. Finally, the calculated scale and the input of SE-Block are multiplied between channels to enhance the important features and weaken the unimportant features, so that the extracted features are more directional.

6 $$\begin{array}{*{20}c} {\tilde{h}_{c} = s_{c} h_{c} } \\ \end{array}$$

### Aggregation and pooling

X-vector and some ResNet-based models set the output features of the last frame-level layer as the input of the pooling layer. These models extract speaker embeddings from one frame-level feature, which results in many shallow layer features are discarded. The shallow features contain a lot of texture information, which are beneficial to robust speaker embeddings. The deep features have a large receptive field, which have a strong ability to represent semantic information. In order to use the complementary features of deep and shallow layers and inspired by Soonshin's work^32^, this paper designs multi-layer feature aggregation to explore straticulate time–frequency interaction. The frame-level embeddings at each Lambda-SE module are concatenated using multi-layer features aggregation to establish shortcut connections from low-level and mid-level features to the final speaker embeddings. As shown in Fig. 2, the input of each Lambda-SE module is obtained by adding the input and output of the previous module, and the calculation process is as shown in Eq. (7). After calculating the outputs of the three Lambda-SE modules, we spliced them together for multi-layer feature aggregation. Then converted frame-level features into segment-level features through attentive statistical pooling.

7 $$\begin{array}{*{20}l} {\left\{ {\begin{array}{*{20}l} {v_{1} = LambdaSE_{1} \left( x \right)} \\ {v_{2} = LambdaSE_{2} \left( {x + v_{1} } \right)} \\ {v_{3} = LambdaSE_{3} \left( {x + v_{1} + v_{2} } \right)} \\ {v = concatenate\left( {v_{1} ,v_{2} ,v_{3} } \right)} \\ \end{array} } \right.} \\ \end{array}$$

where, $x$ is the frame-level feature after the first convolutional layer has increased the dimension, ${v}_{1}, {v}_{2}, {v}_{3}$ are the outputs of the three Lambda-SE modules, and ${v}_{1}, {v}_{2}, {v}_{3}$ have the same data dimension, they will be connected together in the temporal dimension to acquire multi-layer features. As shown in Table 1, for the sake of increasing the acquisition of global information, we connect the features after dimensionality exaltation by the convolutional layer with its mean $\mu$ and standard deviation $\sigma$.

8 $$\begin{array}{*{20}c} {v^{\prime} = concatenate\left( {v,\mu ,\sigma } \right)} \\ \end{array}$$

Frame-level features are aggregated into segment-level features by attentive statistical pooling^33^ operation, which yields an importance weighting of the mean and standard deviation by attention.

9 $$\begin{array}{*{20}c} {e_{t} = v^{{\text{T}}} f\left( {Wv_{t}^{^{\prime}} + b} \right) + k} \\ \end{array}$$

where $f(\cdot )$ is a non-linear activation function ReLU. Normalize the weights using the softmax function:

10 $$\begin{array}{*{20}c} {\alpha_{t} = \frac{{\exp \left( {e_{t} } \right)}}{{\mathop \sum \nolimits_{\tau }^{T} \exp \left( {e_{\tau } } \right)}}} \\ \end{array}$$

The weighted standard deviation is defined as Eq. (11), which enables speaker embeddings to capture the long-term change of speakers more accurately and effectively by taking advantage of attention and statistical pooling. Where $\widetilde{\mu }$ is the weighted mean vector, which is obtained by multiplying the normalized weight ${\alpha }_{t}$ and ${v}_{t}^{^{\prime}}$.

11 $$\begin{array}{*{20}c} {\tilde{\sigma } = \sqrt {\mathop \sum \limits_{t}^{T} \alpha_{t} v_{t}^{^{\prime}} \odot v_{t}^{^{\prime}} - \tilde{\mu } \odot \tilde{\mu }} } \\ \end{array}$$

## Experiments

### Dataset

In this experiment, the Voxceleb1^34^ and the Voxceleb2^35^ training set will be used for model training, and the Voxceleb1 test set will be applied to evaluate performance. They are large-scale text-independent datasets and widely used in speaker verification tasks. Their details are shown in Table 2. The Voxceleb1 dataset consists of 153,357 utterances from 1251 speakers, 148,642 utterances samples from 1211 speakers in the training set, and 4715 utterances combinations from the remaining 40 speakers in the test set to form 37,720 pairs of test pairs. The Voxceleb2 training set contains 1,092,009 utterances from 5994 speakers.

**Table 2:** Details of Voxceleb1 and Voxceleb2 datasets.

Dataset	Voxceleb1	Voxceleb2
Total speakers	1251	6112
Dev speakers	1211	5994
Test speakers	40	118
Total utterances	153,357	1,128,246
Dev utterances	148,642	1,092,009
Test utterances	4715	36,237
Size(h)	352	2442
Avg length of utterances(s)	8.2	7.8

### Data augmentation

Data augmentation has always been a significant research direction in the field of deep learning. This technology can eliminate the phenomenon of overfitting caused by insufficient data volume. It increases the number and diversity of data and improves the robustness of the model.

In the experiment, we enhance the generalization ability of the model by adding additive noise and reverberation to the original Voxceleb dataset, these noise data were obtained from the MUSAN dataset^36^ and the room impulse responses (RIRs) dataset^37^. The MUSAN dataset consists of about 109 h of music, speech, and noise.

- Music includes 42 h of music recordings of different genres.
- Speech has 60 h of recordings from 12 languages, mainly speeches from the public domain, hearings, and debates.
- Noise contains 929 various noises with a total duration of about 6 h, including dial tone, fax machine noise, etc.

During training, we fuse raw audio with one of the following six strategies for data augmentation, including time shift augmentation, RIRs, music, noise, speech and music + speech. Time shift augmentation is a random roll within ± 5% of the time axis, and wraparound transitions to preserve all information. Signal-to-noise ratio (SNR) of the additive noises is stochastically choose in the range of 5-15 dB for the music, 13-20 dB for the speech, and 0-15 dB for the noise. For reverberation, we superimpose the audio signal and the audio of RIR characteristic of reverberation in space to simulate far-field sound. The amount of data after data enhancement is twice as large as the original dataset.

### Training settings

The network model input features are from 80-dimensional MFCCs with a frame length of 25 ms and a frame shift of 10 ms. In the training phase, we fixed the length of the input sequence to 200 frames. While in the testing phase, we fed the full-length utterances into the model. The experiment uses an SGD optimizer with a momentum of 0.99. To prevent overfitting, train with a decaying learning rate, the initial learning rate is set to 0.001 and the decaying weight is set to 0.97. We train Lambda-vector for 80 epochs on the development sets of Voxceleb1 and Voxceleb2 respectively. After each training round is completed, the trained models are evaluated for performance in the Voxceleb1 test set. To obtain more discriminative speaker embeddings, we use the additive angular margin softmax (AAM-Softmax) loss^38^:

12 $$\begin{array}{*{20}c} {L = - \frac{1}{N}\mathop \sum \limits_{i = 1}^{N} log\frac{{e^{{s\left( {\cos \left( {\theta_{{y_{i} }} + m} \right)} \right)}} }}{{e^{{s\left( {\cos \left( {\theta_{{y_{i} }} + m} \right)} \right)}} + \mathop \sum \nolimits_{{j \ne y_{i} }} e^{{s \cdot cos\theta_{j} }} }}} \\ \end{array}$$

where $s$ is a scaling factor and $m$ is the margin. In this experiment, $s$ is set to 30 and $m$ is set to 0.2. The scores for the register voice and the test voice are generated by computing cosine similarity using the speaker embeddings. Equal error rate (EER) and minimum detection cost function (minDCF) are set as performance evaluation metrics. EER is the error rate when false alarm rate (FAR) and false reject rate (FRR) are equal. This leads to $EER=FAR=FRR$. The calculation formula of minDCF is as follows:

13 $$\begin{array}{*{20}c} {minDCF = C_{fa} \times FAR \times \left( {1 - p_{target} } \right) + C_{fr} \times FRR \times p_{target} } \\ \end{array}$$

where, ${C}_{fa}$ is the risk coefficient of wrongly alarming samples, ${C}_{fr}$ is the risk coefficient of wrongly rejecting samples, ${p}_{target}$ is the prior probability of positive example pairs, and $1-{p}_{target}$ is the prior probability of negative example pairs. In this experiment, ${p}_{target}$ is set to 0.01, and equal weights of 1.0 between ${C}_{fa}$ and ${C}_{fr}$. The smaller the EER and minDCF, the better the performance of the model. The current speaker verification systems that the research index is still dominated by EER, and this paper also mainly analyzes the EER results, with minDCF assisting.

## Results

Table 3 lists the experimental results of the Lambda-vector model and some current speaker verification models trained on the Voxceleb1 and Voxceleb2 datasets, and tested on Voxceleb1 dataset. From the table above we can see that the performance of ResNet-based network models is generally better than x-vector. They are better that the embedding features extracted by the deep network model. The performance of the Lambda-vector is better than that of x-vector and most ResNet-based models. The EER of Lambda-vector on Voxceleb1 has a 59.77% reduction compared to x-vector, and a reduction of 21.84–30.92% compared to the ResNet-besed models. On Voxceleb2 dataset, the reduction of EER came to 70.90% and 20.21–52.59% respectively. In summary, our Lambda-vector with Lambda-SE modules and multi-layer feature aggregation currently maintains the progressive result on the Voxceleb1 and Voxceleb2 dataset.

**Table 3:** Comparison of the proposed method with the existing research on Voxceleb1 test set.

Model	Dim	Training set	EER	minDCF(0.01)
x-vector^20^	512	Voxceleb1	7.83%	–
ResNet-20^39^	128		4.30%	0.413
ResNet-34^29^	256		4.03%	0.402
ResNet-34^40^	128		4.56%	0.441
H-vector^41^	512		4.28%	–
Ours	512		3.15%	0.325
x-vector^20^	512	Voxceleb2	7.87%	–
ResNet-34^35^	512		4.83%	–
ResNet-50^35^	2,048		3.95%	–
ResNet-50^42^	2,048		2.94%	0.278
Thin ResNet-34^43^	512		2.87%	0.31
Thin ResNet-34^44^	512		3.22%	–
H-vector^41^	512		3.21%	–
Ours	512		2.29%	0.244

Dim represents the dimension of the embedding feature.

To further test the performance of the proposed model, we completed experiments on the x-vector^15^ and ResNet-SE^24,45^ models. ResNet-SE is mainly obtained by adding SE-Block on the basis of ResNet. Table 4 shows experimental results of x-vector, 34 layers of ResNet-SE and Lambda-vector on the Voxceleb1 dev and test set. The parameter amount of Lambda-vector is 82.85% of that of x-vector, but its EER is reduced by 58.93%. The number of parameters of Lambda-vector is less than one-sixth of ResNet-SE, but its EER is 11.52% lower than ResNet-SE. It achieves better performance with lower computational complexity. The model extracts more abundant speaker embeddings than traditional networks due to the nuanced learning ability of Lambda-SE and the strong capture ability of different layer features of multi-layer feature aggregation. The temporal horizon of the x-vector system is limited to 15 frame-level features. The limited temporal context of frame-level features is not enough and should be supplemented with global-based corpus information. Compared with x-vector, Lambda-vector can extract long-distance interaction and affluent global information.

**Table 4:** Results of x-vector, ResNet-SE and Lambda-vector models on Voxceleb1 test set. M for Million.

Model	Params	EER	minDCF(0.01)
x-vector	5.89 M	7.67%	0.584
ResNet-SE	26.8 M	3.56%	0.387
Lambda-vector	4.88 M	3.15%	0.325

The reason Lambda-vector model has achieved better results than ResNet-based methods may be that the multi-layer feature aggregation structure integrates the frame-level features of different network layers, so that the speaker embeddings have more local information. Shallow features contain more global information, which means that contributions from different regions in the utterances will be balanced. Deep features are very useful for learning speaker-related information within a specific region of utterances and reducing possible interference from other regions. It also fused the mean vector and standard deviation of the frame-level features to have more global information, which improves the discrimination of embedding features.

**Figure 5:**
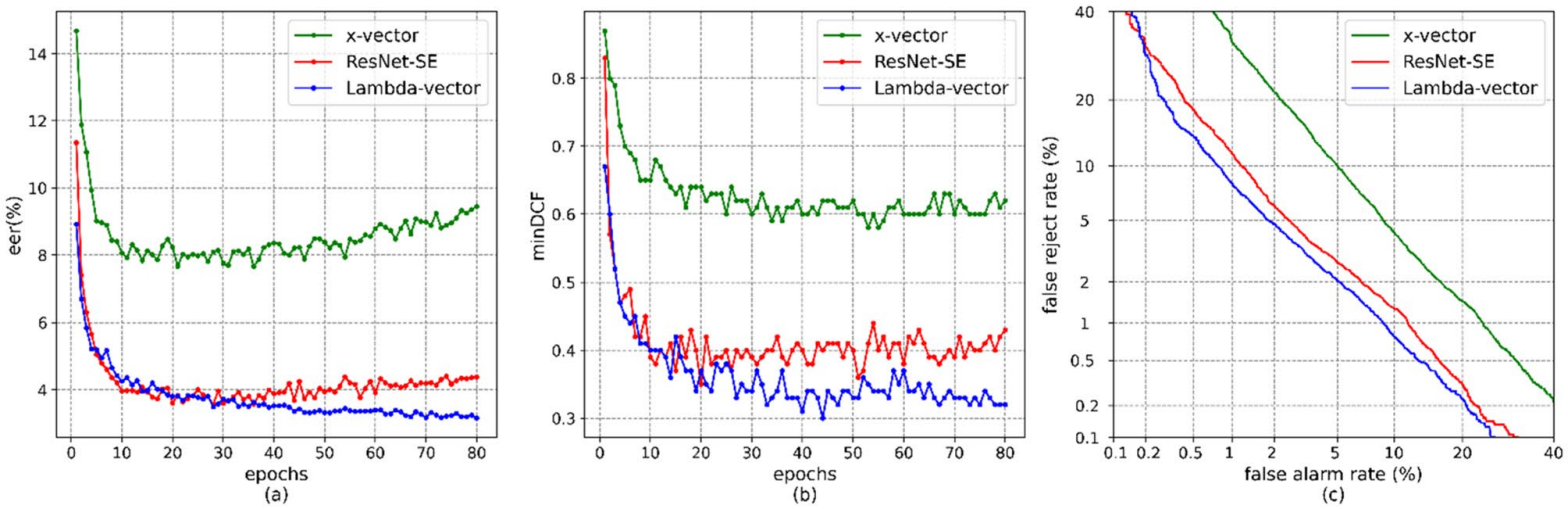
(**a**) EER changes with different training rounds. (**b**) minDCF changes with different training rounds. (**c**) The DET curve of the three models on Voxceleb1 test set.

Figure 5a,b show the EER and minDCF curves of the x-vector, ResNet-SE and Lambda-vector during 80 rounds of training. The EER and minDCF of all models decreased with epochs, and the value of EER and minDCF rapid decline in rounds 0–10, slow change in rounds 10–80. We found that the EER and minDCF trends of these models are not the same, which is due to that minDCF further considers the prior probabilities and different costs on top of the EER. The initial value and result of Lambda-vector are the lowest among the three models. Compared with x-vector and ResNet-SE, the EER of Lambda-vector decreases steadily throughout the training process, while the EER of x-vector and ResNet-SE have relatively large fluctuations and a slight upward trend. Figure 5c shows the detection error tradeoff (DET) curves of the x-vector, ResNet-SE and Lambda-vector on the Voxceleb1 test set. It can be seen from the figure, most of the FAR and FRR obtained by x-vector are the highest. ResNet-SE achieves higher FAR and EER, but still lower than that obtained by the x-vector. This is due to the deep network of ResNet-SE, which has better nonlinear expression ability and can learn more complex transformations to fit complex features. Lambda-vector achieves the lowest EER and FAR on account of the contributions of Lambda-SE and multi-layer feature aggregation. At the same time, we also found that the FRR of Lambda-vector is higher than that of ResNet-SE, which indicates that Lambda-vector will treat the recognition results more prudently.

**Figure 6:**
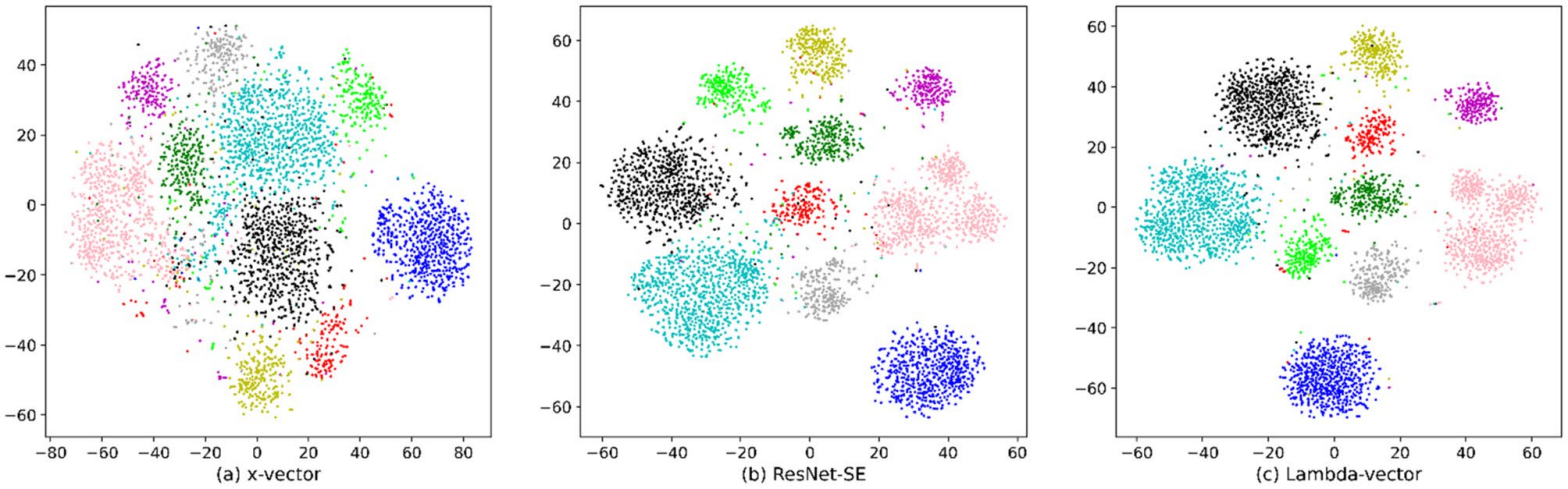
Speaker embedding visualization of x-vector, ResNet-SE and Lambda-vector using t-SNE in the Voxceleb1 test set. Each color represents a speaker, and each point represents a segment with a length of 1 s of audio.

To evaluate the quality of the embedding features generated by the three models, this experiment selects 10 speakers’ utterances from the Voxceleb1 test set, intercepts these utterances into audio segments with lengths of many 1 s to extract the embedding features, and uses t-SNE^46^ to visualize embeddings. The 512-dimensional speaker embeddings are reduced to 2 dimensions through t-SNE, and then projected onto the 2D plane to illustrate the distribution of embeddings. Figure 6a–c show the t-SNE visualization of x-vector, ResNet-SE, and Lambda-vector. Each color represents a speaker, and each point represents a segment with a length of 1 s of audio. From the figure, it can be found that the t-SNE overlap of x-vector is large, the intra-class distance is large, the inter-class distance is small, and the quality of the generated embedding features is the worst. The intra-class distance of Lambda-vector and ResNet-SE is not much different, but the inter-class distance of Lambda-vector is larger. The focus of ResNet-SE is to model channel-related interactions, while Lambda-vector models both temporal and channel-related information. It makes the embedding features of Lambda-vector have better discrimination and representation.

**Figure 7:**
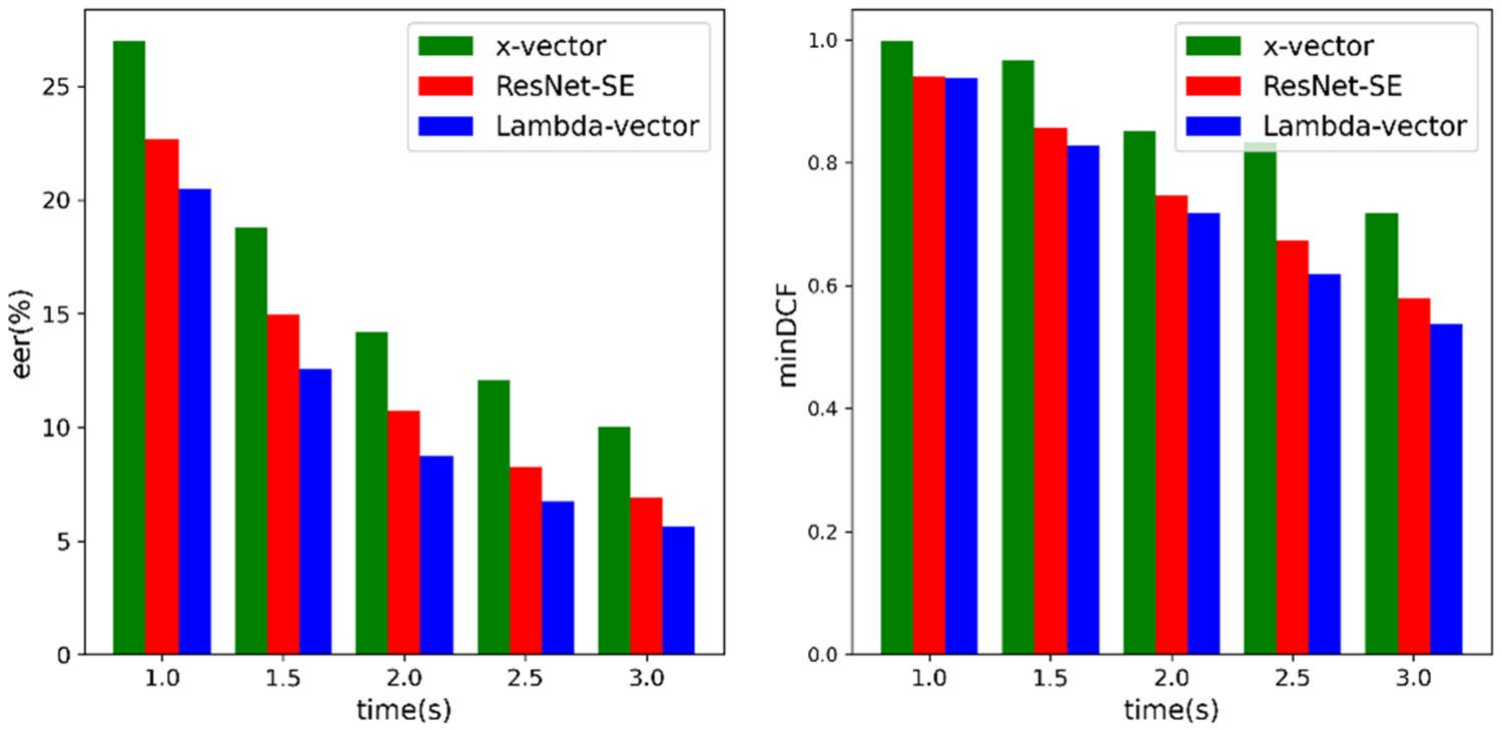
EER and minDCF on Voxceleb1 test set when the utterance length is 1 s, 1.5 s, 2 s, 2.5 s and 3 s.

In the actual use process, there is inevitably the problem of short speech duration, so short-term speaker verification is an important indicator of model evaluation. Figure 7 shows the EER and minDCF performance of x-vector, ResNet-SE and Lambda-vector with speech durations of 1 s, 1.5 s, 2 s, 2.5 s and 3 s. It can be seen that the EER and minDCF of models decreased over time, and the optimal results are obtained when the duration is 3 s. The longer the speech duration, the more speaker characteristics it contains, and the higher the identification accuracy. The EER and minDCF value of Lambda-vector is the lowest at each time length, while the results of x-vector and ResNet-SE are high. Compared with Lambda-vector, the slope of the minDCF of the x-vector fluctuates greatly. The trend suggests that the x-vector with only one layer of frame-level features extracted has poor stability. It also shows that multi-layer features aggregation has improve model performance. When the speech duration of 1 s, the minDCF of Lambda-vector and ResNet-SE are quite close to each other, which may be because the duration is too short for both models to extract enough personal information.

## Conclusion

For the purpose of solving the problems of large amount of deep feature parameters and insufficient feature extraction capability of shallow features, this paper proposes a shallow speaker verification model based on Lambda-vector. It introduces the Lambda-SE modules as the main body of the model, which enable Lambda-vector to simultaneously extract temporal dependencies and channel interactions. Multi-layer feature aggregation is applied to fuse deep features and shallow features, so that embedding features contain more semantic and detailed information and enhance the discrimination of embedding features. We perform data augmentation on the speech from public datasets, Voxceleb1 and Voxceleb2, to obtain a pair of noisy and noise-free utterances, designed to maximize the model's generalization ability. The results on the Voxceleb1 test set show that, compared with x-vector and ResNet-SE, Lambda-vector has fewer parameters, and has different degrees of reduction in EER and minDCF. However, we find that the EER and minDCF of these speaker verification models including Lambda-vector rise significantly when the speech length is less than 2 s. In the next step, we will investigate how to improve the recognition performance of the models under short-time speech conditions.

## Data Availability

The datasets generated during and/or analysed during the current study are available from the corresponding author on reasonable request.

## References

[1] Huang, W., Zhang, Y. & Wan, S. A sorting fuzzy min-max model in an embedded system for atrial fibrillation detection. *ACM Trans. Multimed. Comput. Commun. Appl.* (2022).

[2] Bai Z, Zhang X-L (2021). Speaker recognition based on deep learning: an overview. Neural Netw..

[3] Matsui T, Furui S (1994). Comparison of text-independent speaker recognition methods using VQ-distortion and discrete/continuous HMM’s. IEEE Trans. Speech Audio Process..

[4] Reynolds DA (1995). Speaker identification and verification using Gaussian mixture speaker models. Speech Commun..

[5] Kenny, P. Joint factor analysis of speaker and session variability: Theory and algorithms. *CRIM MontrealReport CRIM-0608–13* **14**, 2 (2005).

[6] Garcia-Romero, D. & Espy-Wilson, C. Y. Analysis of i-vector length normalization in speaker recognition systems. in *interspeech 2011* 249–252 (2011).

[7] Zhang, S.-X., Chen, Z., Zhao, Y., Li, J. & Gong, Y. End-to-end attention based text-dependent speaker verification. in *2016 IEEE Spoken Language Technology Workshop (SLT)* 171–178 (2016).

[8] Cai, W., Chen, J. & Li, M. Analysis of length normalization in end-to-end speaker verification system. in *interspeech 2018* 3618–3622 (2018).

[9] Gao, Z. *et al.* Improving Aggregation and Loss Function for Better Embedding Learning in End-to-End Speaker Verification System. in *interspeech 2019* 361–365 (2019).

[10] Lin, W., Mak, M.-W. & Chien, J.-T. Strategies for End-to-End Text-Independent Speaker Verification. in *interspeech 2020* 4308–4312 (2020).

[11] Ramoji, S., Krishnan, P. & Ganapathy, S. Neural PLDA modeling for end-to-end speaker verification. in *interspeech 2020* 4333–4337 (2020).

[12] Prince, S. J. & Elder, J. H. Probabilistic linear discriminant analysis for inferences about identity. in *2007 IEEE 11th international conference on computer vision* 1–8 (2007).

[13] Variani, E., Lei, X., McDermott, E., Moreno, I. L. & Gonzalez-Dominguez, J. Deep neural networks for small footprint text-dependent speaker verification. in *2014 IEEE international conference on acoustics, speech and signal processing (ICASSP)* 4052–4056 (2014).

[14] Snyder, D., Garcia-Romero, D., Povey, D. & Khudanpur, S. Deep neural network embeddings for text-independent speaker verification. in *interspeech 2017* 999–1003 (2017).

[15] Snyder, D., Garcia-Romero, D., Sell, G., Povey, D. & Khudanpur, S. X-vectors: Robust dnn embeddings for speaker recognition. in *2018 IEEE International Conference on Acoustics, Speech and Signal Processing (ICASSP)* 5329–5333 (2018).

[16] Gou, J. *et al.* Multi-Level Attention-Based Sample Correlations for Knowledge Distillation. *IEEE Trans. Ind. Inform.* 1–11 (2022).

[17] Zhang, Y. *et al.* Local Correlation Ensemble with GCN based on Attention Features for Cross-domain Person Re-ID. *ACM Trans. Multimed. Comput. Commun. Appl.* (2022).

[18] Wu, Y. *et al.* Joint Intent Detection Model for Task-oriented Human-computer Dialogue System using Asynchronous Training. *ACM Trans. Asian Low-Resour. Lang. Inf. Process.* (2022).

[19] Vaswani, A. *et al.* Attention is all you need. *Adv. Neural Inf. Process. Syst.* 5998–6008 (2017).

[20] Safari P, India M, Hernando J (2020). Self-attention encoding and pooling for speaker recognition. Interspeech.

[21] Wang, R. *et al.* Multi-View Self-Attention Based Transformer for Speaker Recognition. *ArXiv Prepr. ArXiv211005036* (2021).

[22] Liu, Z. *et al.* Swin transformer: Hierarchical vision transformer using shifted windows. in *Proceedings of the IEEE/CVF International Conference on Computer Vision* 10012–10022 (2021).

[23] Bello, I. Lambdanetworks: Modeling long-range interactions without attention. in *International Conference on Learning Representations(ICLR)* (2021).

[24] Hu, J., Shen, L. & Sun, G. Squeeze-and-excitation networks. in *Proceedings of the IEEE conference on computer vision and pattern recognition(CVPR)* 7132–7141 (2018).

[25] He, K., Zhang, X., Ren, S. & Sun, J. Deep residual learning for image recognition. in *Proceedings of the IEEE conference on computer vision and pattern recognition(CVPR)* 770–778 (2016).

[26] Yamamoto, H., Lee, K. A., Okabe, K. & Koshinaka, T. Speaker Augmentation and Bandwidth Extension for Deep Speaker Embedding. in *interspeech 2019* 406–410 (2019).

[27] Garcia-Romero, D. *et al.* x-Vector DNN Refinement with Full-Length Recordings for Speaker Recognition. in *interspeech 2019* 1493–1496 (2019).

[28] Snyder, D. *et al.* Speaker Recognition for Multi-speaker Conversations Using X-vectors. in *the IEEE International Conference on Acoustics, Speech and Signal Processing (ICASSP)* 5796–5800 (2019).

[29] Jung, Y., Kim, Y., Lim, H., Choi, Y. & Kim, H. Spatial pyramid encoding with convex length normalization for text-independent speaker verification. in *interspeech 2019* 4030–4034 (2019).

[30] Garcia-Romero, D., Sell, G. & Mccree, A. Magneto: X-vector magnitude estimation network plus offset for improved speaker recognition. in *Proc. Odyssey 2020 the speaker and language recognition workshop* 1–8 (2020).

[31] Qi, J., Guo, W. & Gu, B. Bidirectional Multiscale Feature Aggregation for Speaker Verification. in *interspeech 2021* 71–75 (2021).

[32] Seo, S. *et al.* Shortcut Connections Based Deep Speaker Embeddings for End-to-End Speaker Verification System. in *interspeech 2019* 2928–2932 (2019).

[33] Okabe, K., Koshinaka, T. & Shinoda, K. Attentive statistics pooling for deep speaker embedding. in *interspeech 2018* 2252–2256 (2018).

[34] Nagrani, A., Chung, J. S. & Zisserman, A. Voxceleb: a large-scale speaker identification dataset. in *interspeech 2017* 2616–2620 (2017).

[35] Chung, J. S., Nagrani, A. & Zisserman, A. Voxceleb2: Deep speaker recognition. in *interspeech 2018* 1086–1090 (2018).

[36] Snyder, D., Chen, G. & Povey, D. Musan: A music, speech, and noise corpus. *ArXiv Prepr. ArXiv151008484* (2015).

[37] Ko, T., Peddinti, V., Povey, D., Seltzer, M. L. & Khudanpur, S. A study on data augmentation of reverberant speech for robust speech recognition. in *2017 IEEE International Conference on Acoustics, Speech and Signal Processing (ICASSP)* 5220–5224 (2017).

[38] Liu, Y., He, L. & Liu, J. Large margin softmax loss for speaker verification. in *interspeech 2019* 2873–2877 (2019).

[39] Hajibabaei, M. & Dai, D. Unified hypersphere embedding for speaker recognition. *ArXiv Prepr. ArXiv180708312* (2018).

[40] Cai, W., Chen, J. & Li, M. Exploring the encoding layer and loss function in end-to-end speaker and language recognition system. in *The Speaker and Language Recognition Workshop* 74–81 (2018).

[41] Shi Y, Huang Q, Hain T (2021). H-VECTORS: Improving the robustness in utterance-level speaker embeddings using a hierarchical attention model. Neural Netw..

[42] Yu, Y.-Q., Fan, L. & Li, W.-J. Ensemble additive margin softmax for speaker verification. in *IEEE International Conference on Acoustics, Speech and Signal Processing (ICASSP)* 6046–6050 (2019).

[43] Nagrani A, Chung JS, Xie W, Zisserman A (2020). Voxceleb: Large-scale speaker verification in the wild. Comput. Speech Lang..

[44] Xie, W., Nagrani, A., Chung, J. S. & Zisserman, A. Utterance-level aggregation for speaker recognition in the wild. in *IEEE International Conference on Acoustics, Speech and Signal Processing (ICASSP)* 5791–5795 (2019).

[45] Zhou, J., Jiang, T., Li, Z., Li, L. & Hong, Q. Deep Speaker Embedding Extraction with Channel-Wise Feature Responses and Additive Supervision Softmax Loss Function. in *interspeech 2019* 2883–2887 (2019).

[46] Van der Maaten L, Hinton G (2008). Visualizing data using t-SNE. J. Mach. Learn. Res..

